# Supplementary material for: Development of a PCR Assay for the Detection of Legionella micdadei in the Environment
Source: Infect Dis Rep. 2025 Oct 17;17(5):131. doi: 10.3390/idr17050131 (PMC12564405; doi:10.3390/idr17050131)
Supplement: Supplementary file 1 [file idr-17-00131-s001.zip › idr-3815294-supplementary.pdf]

infectious disease reports

Supplemental Table S1: Bacterial strains and other specimens used in this study.

Species	Strain	Isolation		gDNA/rxn	PCR (Ct)		
		Source	Year		*L. micdadei*	Int. ctrl	PCR Int.
Aeromonas caviae	CCRI-14843	Water	2003	1ng	ND	32.60	ND
Aeromonas hydrophila	ATCC 7966	Milk	Unk	1ng	ND	30.80	ND
Alcaligenes faecalis	ATCC 15554	Feces	Unk	1ng	ND	24.44	ND
Aquabacterium citratiphilum	DSM 11900	Water filter	Unk	1ng	ND	32.55	ND
Aquabacterium parvum	DSM 11968	Water filter	Unk	1ng	ND	32.79	ND
Aspergillus flavus	CCUG 28296	Shoe sole	2006	1ng	ND	32.63	ND
Aspergillus fumigatus	CCUG 35874	Human	2006	1ng	ND	32.93	ND
	ATCC 90906	Human	2006	1ng	ND	33.23	ND
Aspergillus niger	CCRI-16736	Radio set	Unk	1ng	ND	29.37	ND
Bacillus amyloliquefaciens	CCRI-22546	Unk	Unk	Lysate	ND	29.47	ND
Bacillus atrophaeus	CCRI-9827	Unk	Unk	1ng	ND	20.47	ND
Bacillus cereus	ATCC 14579	Unk	Unk	1ng	ND	32.49	ND
	ATCC 13472	Unk	Unk	1ng	ND	33.20	ND
Bacillus megaterium	ATCC 14581	Unk	Unk	1ng	ND	32.79	ND
Bacillus mycoides	ATCC 6462	Soil	Unk	1ng	ND	32.53	ND
Bacillus subtilis	CCRI-21428	Unk	2011	1ng	ND	32.53	ND
Bacillus thuringiensis	ATCC 10792	Tissue	Unk	1ng	ND	32.34	ND
Burkholderia cepacia	ATCC 25416	Onion	Unk	1ng	ND	30.62	ND
Candida albicans	ATCC 66027	Unk	Unk	1ng	ND	32.33	ND
Candida dubliniensis	NCPF 3949	Human	1998	1ng	ND	32.29	ND
Chlamydophila pneumoniae	ATCC VR1360	Human	Unk	1ng	ND	29.31	ND
Clostridium acetobutylicum	ATCC 824	Cornmeal	Unk	1ng	ND	30.44	ND
Clostridium beijerinkii	ATCC 8260	Unk	Unk	1ng	ND	30.72	ND
Clostridium difficile	ATCC 9689	Unk	Unk	1ng	ND	32.62	ND
Clostridium perfringens	ATCC 13124	Bovine	Unk	1.8ng	ND	32.78	ND
Corynebacterium bovis	ATCC 7715	Milk	Unk	1ng	ND	32.79	ND
Corynebacterium jeikeium	CCRI-10257	Human	Unk	1ng	ND	32.88	ND
Coxiella burnetti	Nine Mile phase II	Unk	Unk	1ng	ND	29.18	ND
Enterococcus faecalis	ATCC 29212	Human	Unk	1ng	ND	32.21	ND
Enterococcus faecium	ATCC 19434	Unk	Unk	1ng	ND	32.24	ND
Escherichia coli	ATCC 11775	Human	Unk	1ng	ND	30.41	ND
Flavobacterium johnsoniae	CCRI-10658	Cow	1981	Lysate	ND	29,97	ND
Hafnia alvei	ATCC 13337	Unk	Unk	1ng	ND	32.31	ND
Homo sapiens	N/A	Human	N/A	1ng	ND	31.10	ND
	N/A	Human	N/A	1ng	ND	30.81	ND
	N/A	Human	N/A	1ng	ND	30.65	ND
Klebsiella aerogenes	ATCC 13048	Human	Unk	1ng	ND	30.72	ND
Klebsiella oxytoca	ATCC 13182	Human	Unk	1ng	ND	30.68	ND
Legionella anisa	CCRI-25814	Env	2024	Lysate	ND	32.23	ND

	CCRI-25815	Env	2024	Lysate	ND	32.52	ND
	CCRI-25816	Env	2024	Lysate	ND	32.73	ND
	CCRI-25842	Water	Unk	Lysate	ND	29.04	ND
Legionella bozemanae	ATCC 33217	Human	2005	1ng	ND	32.43	ND
	ID108728	Human	Unk	Lysate	ND	29.13	ND
Legionella dumoffii	CCRI-25821	Env	2021	Lysate	ND	32.39	ND
Legionella gormanii	L00153453	Human	Unk	Lysate	ND	29.24	ND
Legionella jordanis	CCRI-11729	River Water	Unk	1ng	ND	32.83	ND
Legionella longbeachae	L00659637	Human	Unk	Lysate	ND	29.33	ND
Legionella micdadei	CCRI-25807	Human	Unk	10cp	33.10	32.80	D
	CCRI-25808	Human	2024	10cp	33.10	32.60	D
	CCRI-25809	Human	2024	10cp	33.70	32.70	D
	CCRI-25810	Human	2024	10cp	33.50	32.80	D
	CCRI-25817	Human	2024	10cp	35.00	32.70	D
	CCRI-25819	Human	2023	10cp	32.20	32.70	D
	CCRI-25820	Human	2023	10cp	33.00	32.50	D
	ATCC 33218	Human	1980	10cp	32.20	32.95	D
	ID063037	Human	Unk	Lysate	24.30	-	D
	ID081869	Human	Unk	Lysate	24.70	-	D
	ID088926	Human	Unk	Lysate	25.20	32.42	D
	ID090456	Human	Unk	Lysate	25.10	30.35	D
	ID103059	Human	Unk	Lysate	24.50	-	D
	ID108784	Human	Unk	Lysate	24.80	-	D
	ID114570	Human	Unk	Lysate	25.10	33.40	D
	ID125965	Human	Unk	Lysate	25.30	-	D
	L00132833	Human	Unk	Lysate	25.20	33.60	D
	L00166407	Human	Unk	Lysate	25.10	32.39	D
	L00495253	Human	Unk	Lysate	25.10	33.25	D
	CCRI-25845	Human	Unk	Lysate	24.60	-	D
Legionella pneumophila	ATCC 33152	Human	Unk	1ng	ND	32.79	ND
	ATCC 33215	Human	Unk	Lysate	ND	32.34	ND
	CCRI-25844	Water	Unk	Lysate	ND	29.26	ND
Legionella quinlivanii	ID143958	Human	Unk	Lysate	ND	29.16	ND
Legionella sp.	CCRI-25841	Water	Unk	Lysate	ND	29.16	ND
	CCRI-25843	Water	Unk	Lysate	ND	29.24	ND
Legionella yabuuchiae	L00683472	Human	Unk	Lysate	ND	29.07	ND
Listeria monocytogenes	ATCC 15313	Rabbit	1924	1ng	ND	29.88	ND
Methylobacterium aminovorans	ATCC 51358	Soil	Unk	1ng	ND	32.88	ND
	CCRI-9043	Soil	Unk	1ng	ND	29.36	ND
Moraxella catarrhalis	ATCC 43628	Human	Unk	Lysate	ND	32.65	ND
Mycobacterium avium	ATCC 25291	Chicken	Unk	1ng	ND	32.5	ND
Mycobacterium smegmatis	CCRI-5439	Unk	Unk	1ng	ND	33.25	ND
Pantoea agglomerans	ATCC 43875	Unk	Unk	1ng	ND	32.51	ND
Plesiomonas shigelloides	ATCC 14029	Unk	Unk	1ng	ND	32.35	ND
Proteus vulgaris	ATCC 29905	Unk	Unk	1ng	ND	30.60	ND
Pseudomonas aeruginosa	CCRI-701	Unk	Unk	1ng	ND	32.39	ND
	CCRI-1236	Human	Unk	1ng	ND	32.42	ND
	CCRI-12850	Unk	Unk	1ng	ND	32.76	ND

Pseudomonas fluorescens	CCRI-705	Unk	Unk	1ng	ND	32.45	ND
Pseudomonas oryzihabitans	ATCC 43272	Rice	Unk	1ng	ND	32.87	ND
Pseudomonas putida	CCRI-21588	Unk	2011	1ng	ND	32.63	ND
Pseudomonas stutzeri	ATCC 17588	Unk	Unk	1ng	ND	32.61	ND
Rhodococcus equi	ATCC 6939	Foal	Unk	1ng	ND	32.64	ND
Serratia ficaria	ATCC 33105	Fig	Unk	1ng	ND	32.57	ND
Serratia liquefaciens	ATCC 27592	Milk	Unk	1ng	ND	32.75	ND
Serratia marcescens	CCRI-791	Unk	Unk	1ng	ND	32.79	ND
Shewanella putrefaciens	CCRI-2019	Unk	Unk	Lysate	ND	29.23	ND
Sphingomonas paucimobilis	ATCC 29837	Unk	Unk	1ng	ND	32.2	ND
Staphylococcus aureus	ATCC 43300	Human	Unk	1ng	ND	32.41	ND
Staphylococcus epidermidis	ATCC 14990	Human	Unk	1ng	ND	32.4	ND
Stenotrophomonas maltophilia	CCRI-751	Human	Unk	1ng	ND	32.95	ND
Streptococcus pneumoniae	ATCC 6303	Human	1994	1ng	ND	32.37	ND
Streptococcus pyogenes	ATCC 19615	Human	Unk	1ng	ND	32.35	ND
N/A	Blank swab	N/A	N/A	Lysate	ND	33.08	ND

Abbreviations: rxn = reaction; ; Int. ctrl = internal control; PCR int. = PCR interpretation; D = detected; ND = not detected; Unk = unknown; Env = environment; N/A = not applicable.
